# Healthy Behavior: The Truth

**Published:** 2008-06-15

**Authors:** Lynne S. Wilcox

What more do we need to know? We've defined the basics — eat right, exercise, don't smoke, get screened. Follow these rules and your risk for chronic diseases and their complications may decline by 25% or more.

But as Oscar Wilde wrote, "The pure and simple truth is rarely pure and never simple" (1). This issue of *Preventing Chronic Disease* highlights our struggles to find the truth and promote health amid all the world's influences. We have learned that telling people "what's good for them" is rarely enough to ensure healthy habits. Even in-depth individual coaching may be insufficient. The family kitchen, the community grocery store, the school, the workplace, the nation — all affect behavior. The good news is that we are recognizing and investigating this complexity.

The reports in this issue can be understood in terms of the classic cycle of public health systems: 1) data collection, research, and analysis; 2) evidence-based policy development; 3) programs derived from policy; and 4) program evaluation and feedback to data collection systems.

Articles in this issue discuss 2 kinds of data collection: large, population-based surveillance reports and methods and smaller community surveys or focus groups. In the first group are articles about surveillance methods (2-6), physical activity in Mississippi (7), chronic disease in Southeast Asia (8), U.S. tobacco use (9), and trends in chronic disease prevalence in Oman (10). We also report on adolescent obesity in California (11), trends in hepatocellular carcinoma in the United States (12), trends in gestational diabetes and pregnancy-related hypertension in Los Angeles County, California (13), and substance use in Addis Ababa, Ethiopia (14).

The second group of data collection articles includes several focus group reports: young adults on use of nontraditional tobacco products (15), college students at a historically black university on use of little cigars (16), Arab Israeli college students on physical activity (17), and Samoan adults with diabetes on perceptions of their disease (18). What a diversity of data sources, populations, and topics, all to provide an evidence base for sound policy and programs on healthy behaviors!

Policy is also represented in this issue. Watson and Dannenberg calculate the size and population density of communities most likely to benefit from the Safe Routes to School Program (19). Mbulo examines Nebraska students' continued exposure to secondhand smoke despite smoke-free policies and other efforts (20), and Davison et al review the literature and quality of research on programs that promote active commuting to school (21).

Many of the articles in this issue illustrate the close connection between implementing and evaluating health behavior programs, for example, a pilot study of American Cancer Society Workplace Solutions (22), a joint-use project between Honolulu's schools and its parks service to allow use of school grounds for community recreational activities (23), financial incentives for weight loss in rural Mexican adults (24), and use of peer educators to promote healthy behavior among students in São Paulo, Brazil (25). Matson Koffman et al present a literature review of interventions for high blood pressure and high cholesterol in health care settings (26), and Allen et al describe a group-discussion intervention among American Indian women with impaired fasting blood glucose (27).

In the sense of these multiple perspectives, the truth is indeed intricate. And yet Galileo, a man experienced in difficult truths, said, "All truths are easy to understand once they are discovered; the point is to discover them." After decades of research, observation, and intervention, our basic truth is that healthy behaviors are essential for well-being. Our challenge is to learn how to implement this knowledge for the benefit of every individual and community.
